# Diagnostic accuracy of cross-sectional and endoscopic imaging in ampullary tumours: systematic review

**DOI:** 10.1093/bjs/znad432

**Published:** 2024-01-10

**Authors:** Anouk J de Wilde, Evelien J M de Jong, Kurinchi S Gurusamy, Mohammad Abu Hilal, Marc G Besselink, Maxime J L Dewulf, Sandra M E Geurts, Ulf P Neumann, Steven W M Olde Damink, Jan-Werner Poley, Vivianne C G Tjan-Heijnen, Judith de Vos-Geelen, Georg Wiltberger, Mariëlle M E Coolsen, Stefan A W Bouwense

**Affiliations:** Department of Surgery, Maastricht University Medical Centre+, Maastricht, the Netherlands; Department of Internal Medicine, Division of Medical Oncology, Maastricht University Medical Centre+, GROW, Maastricht University, Maastricht, the Netherlands; Division of Surgery and Interventional Science, Royal Free Campus, University College London, London, UK; Department of Surgery, Fondazione Poliambulanza, Brescia, Italy; Amsterdam UMC, location University of Amsterdam, Department of Surgery, Amsterdam, the Netherlands; Cancer Centre Amsterdam, Amsterdam, the Netherlands; Department of Surgery, Maastricht University Medical Centre+, Maastricht, the Netherlands; Department of Internal Medicine, Division of Medical Oncology, Maastricht University Medical Centre+, GROW, Maastricht University, Maastricht, the Netherlands; Department of Surgery, Maastricht University Medical Centre+, Maastricht, the Netherlands; Department of Surgery, Maastricht University Medical Centre+, Maastricht, the Netherlands; Department of Gastroenterology and Hepatology, Maastricht University Medical Centre+, Maastricht, The Netherlands; Department of Internal Medicine, Division of Medical Oncology, Maastricht University Medical Centre+, GROW, Maastricht University, Maastricht, the Netherlands; Department of Internal Medicine, Division of Medical Oncology, Maastricht University Medical Centre+, GROW, Maastricht University, Maastricht, the Netherlands; Department of General, Visceral, and Transplantation Surgery, University Hospital of RWTH Aachen, Aachen, Germany; Department of Surgery, Maastricht University Medical Centre+, Maastricht, the Netherlands; Department of Surgery, Maastricht University Medical Centre+, Maastricht, the Netherlands

## Abstract

**Background:**

Differentiation between adenomas and carcinomas of the ampulla of Vater is crucial for therapy and prognosis. This was a systematic review of the literature on the accuracy of diagnostic modalities used to differentiate between benign and malignant ampullary tumours.

**Methods:**

A literature search was conducted in PubMed, Embase, CINAHL, and the Cochrane Library. Studies were included if they reported diagnostic test accuracy information among benign and malignant ampullary tumours, and used pathological diagnosis as the reference standard. Risk of bias was assessed using Quality Assessment on Diagnostic Accuracy Studies (QUADAS) 2 and QUADAS-C.

**Results:**

Ten studies comprising 397 patients were included. Frequently studied modalities were (CT; 2 studies), endoscopic ultrasonography (EUS; 3 studies), intraductal ultrasonography (IDUS; 2 studies), and endoscopic forceps biopsy (3 studies). For CT, the reported sensitivity for detecting ampullary carcinoma was 44 and 95%, and the specificity 58 and 60%. For EUS, the sensitivity ranged from 63 to 89% and the specificity between 50 and 100%. A sensitivity of 88 and 100% was reported for IDUS, with a specificity of 75 and 93%. For forceps biopsy, the sensitivity ranged from 20 to 91%, and the specificity from 75 to 86%. The overall risk of bias was scored as moderate to poor. Data were insufficient for meta-analysis.

**Conclusion:**

To differentiate benign from malignant ampullary tumours, EUS and IDUS seem to be the best diagnostic modalities. Sufficient high-quality evidence, however, is lacking.

## Teaser

This study is the first to comprehensively review the available evidence on diagnostic modalities in patients with ampullary tumours. Differentiation between benign and malignant tumours is important in deciding which treatment is needed.

## Introduction

Benign and malignant tumours of the ampulla of Vater (hereafter referred to as ampullary tumours) are relatively rare. For example, in the Netherlands, there were 177 patients with ampullary tumours in 2021 (0.68 per 100 000 in 2010–2016)^1,2^. Benign tumours have a 26– 65% lifetime risk of becoming malignant^3,4^. To differentiate between a benign and malignant tumour, and to select appropriate treatment, clinicians rely on imaging, visual inspection of the tumour during endoscopy, and histological assessment. A clear-cut diagnostic approach to ampullary tumours, however, is lacking.

To assess local characteristics of the tumour (size, location, and depth of infiltration) and its relationship to surrounding tissues (involvement of lymph nodes and vascular structures), multiple diagnostic modalities with different advantages and disadvantages are available, such as abdominal or endoscopic (EUS) ultrasonography, endoscopic retrograde cholangiopancreatography (ERCP), MRI and magnetic resonance cholangiopancreatography, CT, PET–CT, and nuclear scintigraphy^5,6^. In line with the current European Society for Medical Oncology (ESMO) guideline for the assessment of pancreatic and bile duct tumours^7^, EUS and CT are most frequently used of these modalities. Pathological assessment might help to further differentiate between benign and malignant tumours, but sampling errors frequently occur^8,9^. Proper differentiation between benign and malignant ampullary tumours is particularly important in deciding which treatment is needed. For benign ampullary tumours, follow-up (with repeating imaging) or local (endoscopic or surgical) resection is sufficient, whereas oncological resection is preferred if possible for (suspected) malignant tumours^5,6,10–13^.

There is currently no reference standard for the diagnostic approach to ampullary tumours and no previous systematic review on this topic is available^14,15^. The aim of this review was to assess the accuracy of the diagnostic approach to ampullary tumours and, more specifically, the ability to differentiate between benign and malignant tumours.

## Methods

This systematic review was performed according to the Cochrane Handbook and the PRISMA guidelines, and was registered in PROSPERO database (CRD42021269453)^16–19^.

### Search strategy

A systematic search was conducted in the PubMed, Embase, Cumulative Index to Nursing & Allied Health Literature (CINAHL), and the Cochrane Library databases to identify relevant studies assessing the accuracy of the diagnostic procedures for ampullary tumours. These studies included RCTs and (comparative) observational studies. No specific minimum volume was used in this search. The search was performed on 4 February 2022 and included the following search terms: ‘Ampulla of Vater’, ‘Neoplasms’, ‘Common Bile Duct Neoplasm’, ‘Magnetic Resonance Imaging’, ‘Magnetic resonance cholangiopancreatography’, ‘Ultrasonography’, ‘Endoscopic Ultrasound’, ‘Endoscopy, Digestive System’, ‘Endoscopic retrograde cholangiopancreatography’, ‘Tomography, X-Ray Computed’, ‘Duodenoscopy’, ‘PET/CT’, ‘Nuclear scintigraphy’, ‘Cytology’, and ‘Biopsy’. The full search is described in *Appendix S1*. Synonyms of these terms were also used in the search. There were no restrictions on language or publication date. Duplicate references were removed and all search results were uploaded into Rayyan, a web app for filtering eligible studies for a systematic review^20^. If no abstract and/or full-text was available, the authors of the article were contacted by e-mail to obtain them.

### Study selection

All articles were screened by two reviewers independently with respect to the prespecified inclusion and exclusion criteria based on title and abstract. Studies were included if they met the following inclusion criteria: patients had a pathologically confirmed ampullary tumour; the study assessed diagnostic accuracy of a diagnostic modality using histology as the reference standard; and, if non-ampullary tumours were included in the study, diagnostic test accuracy information was available for people with ampullary tumours. Exclusion criteria were study design such as reviews, letters, book chapters, and case reports; and study that included only malignant or only benign tumours. After abstract screening, the two reviewers independently read the full text of potentially useful articles to enable final selection.

### Data extraction and data collection

Two reviewers independently screened all articles, extracted the data, and assessed the risk of bias. Disagreements were resolved by discussion. When no consensus was reached, an arbitrator resolved disagreements. Relevant data were extracted using a data extraction form. Data collected included first author, year of publication, study design (prospective or retrospective cohort study, cross-sectional study, or RCT), total number of patients, patient characteristics (age, sex), number of patients diagnosed with an ampullary tumour (malignant *versus* benign), index diagnostic test, reference test, and diagnostic test accuracy information (true positive, false positive, false negative, and true negative).

### Risk of bias

The Quality Assessment on Diagnostic Accuracy Studies (QUADAS) 2 tool^21^ and QUADAS—Comparative (QUADAS-C) tool^22^ were used to assess the risk of bias. The QUADAS-C is an extension of QUADAS-2 for comparative studies, in which two or more index tests were performed in the same study population. The risk of bias was assessed in four key domains including patient selection, index test(s), reference standard, and flow and timing. Concerns regarding applicability (patient selection, index test(s), and reference standard) were determined. The degree of bias and applicability were expressed as high, low, or unclear, in accordance with the guidance documents.

### Statistical analysis

The statistical analysis was performed using Review Manager 5.3 (RevMan® [Computer program]. Version 5.3. Copenhagen: The Nordic Cochrane Centre, The Cochrane Collaboration, 2014), for generating forest plots. The individual study estimates of sensitivity and specificity were shown as forest plots for the different index tests to examine the variation between studies. Meta-analysis was attempted using SAS® software (SAS Institute Inc., Cary, NC, USA) for calculating the summary sensitivity and specificity. Because of the sparse data, simpler hierarchical models were used for meta-analysis^23^. Visualization of forest plots, and model fit determined by the (-2) log likelihood values, were used to decide on the best model for undertaking meta-analysis. The forest plots of sensitivity and specificity were also inspected visually for potential sources of heterogeneity. Planned subgroup analyses or a meta-regression approach to investigate heterogeneity were not performed because of the sparse data.

## Results

### Selected studies and characteristics

**Fig. 1:**
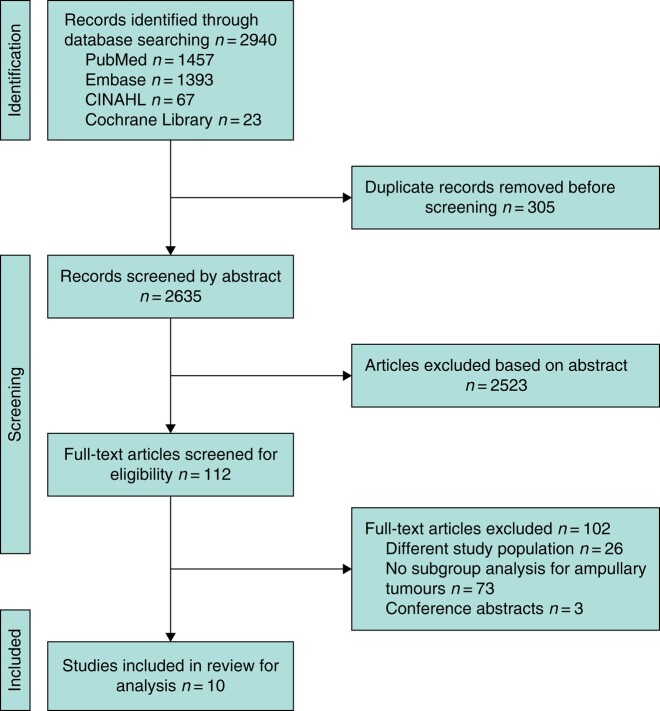
PRISMA flow diagram showing search strategy and study selection. Copyright statement: this PRISMA diagram contains public sector information licensed under the Open Government Licence v3.0. Adapted from: Moher D, Liberati A, Tetzlaff J, Altman DG, The PRISMA Group (2009). Preferred Reporting Items for Systematic Reviews and Meta-Analyses: The PRISMA Statement. PLoS Med 6(6): e1000097. https://doi.org/10.1371/journal.pmed1000097

The search yielded 2940 studies of which 10^24–33^, comprising 397 patients, were included for further analyses (*Fig. 1*). Preliminary results from three conference abstracts^34–36^, which were not published as peer-reviewed articles, were included in the overview of results but not in the analyses because not all diagnostic test accuracy information was presented.

Study characteristics are presented in *Table 1*. All but two studies were conducted retrospectively. The studies by Ito *et al*.^26^ and Menzel *et al*.^28^ were prospective. Included studies were published between 1997 and 2020. The number of patients included ranged from 14 to 118. In total 260 patients (65.5%) had a malignant tumour. The most frequently studied index tests were EUS^27,28,31^ and endoscopic forceps biopsy^26,28,31^, which were both assessed in three studies. IDUS^25,28^, CT^29,32^, and PET–CT^32,33^ were each examined in two studies as index tests. Brush cytology^24^, endoscopic transpapillary biopsy^25^, biopsy obtained by ERCP^30^, side-viewing duodenoscopy^31^, and a combination of CT with MRI^33^ were each investigated in one study. The reference tests in the included studies consisted of pathological assessment of the resection specimen (obtained by surgical, local and/or endoscopic resection; 9 studies) or endoscopic biopsy (1 study). In two studies, some patients had long-term follow-up in the event of a negative biopsy as reference test. The studies did not mention specific symptoms per patient on which the decision was made to perform diagnostic modalities.

**Table 1:** Summary of characteristics of included studies

Reference	Sample size	No. of patients with malignant or benign ampullary lesion	Mean age (years)	Index test	Reference standard
Bardales *et al*.^24^	22	Benign 15 Malignant 7	70	Brush cytology	Histology: 22 biopsy
Heinzow *et al*.^25^	72	Benign 32 Malignant 40	72	ETP IDUS	Histology of surgical resection and long-term-follow-up of negative biopsies*
Ito *et al*.^26^†	40	Benign 8 Malignant 32	65	Endoscopic forceps biopsy	Histology: 30 surgical resections 10 endoscopic resections
Manta *et al*.^27^	24	Benign 5 Malignant 19	60	EUS	Histology: 22 surgical resections 2 endoscopic resections
Menzel *et al*.^28^†	27	Benign 12 Malignant 15	62	IDUS EUS Endoscopic forceps biopsy	Histology: 27 surgical resections
Pongpornsup *et al*.^29^	55	Benign 12 Malignant 43	65	CT	Histology: unclear how obtained
Rodríguez *et al*.^30^	31	Benign 14 Malignant 17	n.r.	Biopsy during ERCP	Histology: 31 surgical resections
Sauvanet *et al*.^31^	26	Benign 4 Malignant 22	57	SVD EUS Endoscopic forceps biopsy	Histology: 24 surgical resections 2 endoscopic resections
Sperti *et al*.^32^	14	Benign 5 Malignant 9	65	CT, [^18^F]FDG PET–CT	Histology: 14 surgical resections
Wen *et al*.^33^	86	Benign 28 Malignant 58	62	[^18^F]FDG PET–CT CT + MRI	Histology: 48 surgical resections 10 biopsies 26 long-term follow-up
**Abstracts only**					
Chen *et al*.^34^	21	n.r.	n.r.	EUS ERCP CT Ultrasonography	Surgical histology
Peng *et al.*^35^	102	Benign 76 Malignant 18 Non-adenomatous 8	60	EUS EUS + biopsy	Surgical or endoscopic histology
Sharaiha *et al*.^36^	58	n.r.	63	EUS EUS + biopsy	Surgical or endoscopic histology

*The proportion of patients for each reference standard is unclear. †Prospective study. ETP, endoscopic transpapillary biopsy; IDUS, intraductal ultrasonography; EUS, endoscopic ultrasonography; n.r., not reported; ERCP, endoscopic retrograde cholangiopancreatography; SVD, side-viewing duodenoscopy; [^18^F]FDG, [^18^F]fluorodeoxyglucose.

### Quality assessment

In general, the studies had a moderate risk of bias according to the QUADAS-2 (*Table S1*). The studies by Manta *et al*.^27^, Rodríguez *et al*.^30^, and Sauvanet *et al*.^31^ were rated as poor regarding patient selection as these studies had inappropriate exclusions and bias was introduced owing to the selection procedure. The reference test in Heinzow *et al*.^25^ was assessed as poor because histopathological confirmation of the final diagnosis was not available for all patients. All studies, except that of Rodríguez *et al*.^30^, were at high risk of bias in the domain flow and timing. This related to different methods of pathology sampling used as reference tests including resection specimen or biopsy, or follow-up of negative biopsies within one cohort. Regarding applicability concerns, Bardales *et al*.^24^ and Sauvanet *et al*.^31^ were scored as poor in terms of patient selection. For all other domains, all studies scored well. Five studies^25,28,31–33^ assessed more than one index test, for which the QUADAS-C tool was used. The risk of bias investigated using QUADAS-C could be interpreted as moderate to poor.

### Diagnostic accuracy

Outcomes reported in the studies are summarized in *Table 2*. The sensitivity and specificity were calculated for each index test. A meta-analysis for each index test with more than two studies was attempted but, owing to the clinical and methodological heterogeneity along with poor overlap of confidence intervals, convergence was obtained only for fixed-effect meta-analysis. This was clearly inappropriate, because of the poor overlap of confidence intervals and so meta-analysis was not undertaken. Only a narrative summary is provided below.

**Table 2:** Summary of diagnostic test accuracy of included studies

Test	Reference	Sample size	Outcomes
CT	Sperti *et al*.^32^	14	TP 4; FP 2; FN 5; TN 3 Sensitivity 44% Specificity 60%
CT	Pongpornsup *et al*.^29^	55	TP 41; FP 5; FN 2; TN 7 Sensitivity 95% Specificity 58%
PET–CT	Sperti *et al*.^32^	14	TP 7; FP 4; FN 2; TN 1 Sensitivity 78% Specificity 20%
PET–CT	Wen *et al*.^33^	86	TP 54; FP 6; FN 4; TN 22 Sensitivity 93% Specificity 79%
EUS	Manta *et al*.^27^	24	TP 17; FP 0; FN 2; TN 5 Sensitivity 89% Specificity 100%
EUS	Menzel *et al*.^28^	16	TP 5; FP 4; FN 3; TN 4 Sensitivity 63% Specificity 50%
EUS	Sauvanet *et al*.^31^	26	TP 16; FP 1; FN 4; TN 5 Sensitivity 80% Specificity 83%
IDUS	Heinzow *et al*.^25^	72	TP 28; FP 3; FN 4; TN 37 Sensitivity 88% Specificity 93%
IDUS	Menzel *et al*.^28^	27	TP 15; FP 3; FN 0; TN 9 Sensitivity 100% Specificity 75%
Forceps biopsy	Ito *et al*.^26^	39	TP 29; FP 1; FN 3; TN 6 Sensitivity 91% Specificity 86%
Forceps biopsy	Menzel *et al*.^28^	27	TP 3; FP 3; FN 12; TN 9 Sensitivity 20% Specificity 75%
Forceps biopsy	Sauvanet *et al*.^31^	26	TP 13; FP 1; FN 7; TN 5 Sensitivity 65% Specificity 83%
CT + MRI	Wen *et al*.^33^	86	TP 52; FP 18; FN 6; TN 10 Sensitivity 90% Specificity 36%
SVD	Sauvanet *et al*.^31^	26	TP 10; FP 0; FN 6; TN 10 Sensitivity 63% Specificity 100%
Brush cytology	Bardales *et al*.^32^	12	TP 7; FP 0; FN 0; TN 5 Sensitivity 100% Specificity 100%
ETP	Heinzow *et al*.^25^	62	TP 22; FP 0; FN 0; TN 40 Sensitivity 100% Specificity 100%
IDUS + ETP	Heinzow *et al*.^25^	72	TP 31; FP 3; FN 1; TN 37 Sensitivity 97% Specificity 93%
Biopsy during ERCP	Rodríguez *et al*.^30^	31	TP 14; FP 3; FN 7; TN 7 Sensitivity 67% Specificity 70%

TP, true positive; FP, false positive; FN, false negative; TN, true negative; EUS, endoscopic ultrasonography; IDUS, intraductal ultrasonography; SVD, side-viewing duodenoscopy; ETP, endoscopic transpapillary biopsy; ERCP, endoscopic retrograde cholangiopancreatography.

#### CT

**Fig. 2:**
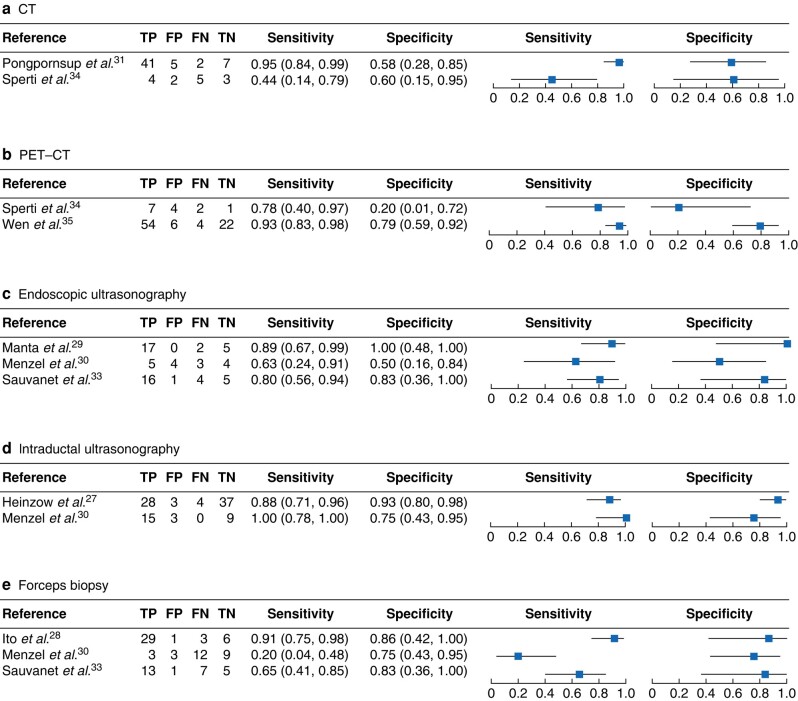
Diagnostic test accuracy of imaging techniques# **a** Diagnostic test accuracy of CT; a random-effects model was used for sensitivity and a fixed-effect model for specificity. **b** Diagnostic test accuracy of PET–CT; a fixed-effect model was used for sensitivity and a random-effects model for specificity. **c** Diagnostic test accuracy for endoscopic ultrasonography; a random-effects model was used for sensitivity and a fixed-effect model for specificity. **d** Diagnostic test accuracy of intraductal ultrasonography; a fixed-effect model was used for sensitivity and a random-effects model for specificity. **e** Diagnostic test accuracy for forceps biopsy; a random-effects model was used for sensitivity and a fixed-effect model for specificity. Point estimates are shown with 95% intervals. TP, true positive; FP, false positive; FN, false negative; TN, true negative.

Two studies reported the sensitivity and specificity of CT. The sensitivity was 44% in Sperti *et al*.^32^ and 95% in Pongpornsup *et al*.^29^. The reported specificity was 58% in Pongpornsup *et al*. and 60% in Sperti *et al*. *Figure 2a* shows the forest plot with corresponding confidence intervals.

#### PET–CT

Sperti *et al*.^32^ and Wen *et al*.^33^ reported a sensitivity of 78% and 93% for PET–CT with a corresponding specificity of 20 and 79% (*Fig. 2b*).

#### Endoscopic ultrasonography

Three studies^27,28,31^ reported on EUS and used pathological resection specimens as reference test. The reported sensitivity was between 63 and 89%, and the specificity between 50 and 100% (*Fig. 2c*).

#### Intraductal ultrasonography

Two studies reported the results of IDUS, compared with the pathology of resection specimens. Heinzow *et al*.^25^ reported a sensitivity of 88% and a specificity of 93%. Menzel *et al*.^28^ reported a sensitivity of 100% and a specificity of 75% (*Fig. 2d*).

#### Forceps biopsy

Results for forceps biopsy were reported in three studies^26,28,31^. The biopsies were compared with the pathology of resection specimens. The sensitivity ranged between 20 and 91%, and the specificity from 75 to 83% (*Fig. 2e*).

#### Additional index tests

Five different index tests were each reported only once, in five different studies. Bardales *et al*.^24^ reported a sensitivity and specificity of 100% for brush cytology. Endoscopic transpapillary biopsy also had a sensitivity and specificity of 100% according to Heinzow *et al*.^25^. The sensitivity and specificity of biopsy during ERCP were 67 and 70% respectively^30^. For side-viewing duodenoscopy, Sauvanet *et al*.^31^ reported a sensitivity of 63% and a specificity of 100%, whereas Wen *et al*.^33^ reported a sensitivity of 90% and specificity of 36% for CT + MRI.

The diagnostic test accuracy reported in the conference abstracts^34–36^, which could not be analysed owing to missing diagnostic test accuracy information, is listed in *Table S2*.

## Discussion

This systematic review of the diagnostic approach to assessment of benign and malignant ampullary tumours showed wide variation in diagnostic modalities currently being used in daily clinical practice. EUS and IDUS seem to have the best sensitivity and specificity. A meta-analysis could not be performed because of the limited amount of data, clinical and methodological heterogeneity, and poor overlap of confidence intervals between the studies. Therefore, no firm conclusions can be drawn on which diagnostic modality is best for assessing the nature of ampullary tumours.

Specific guidelines on how to diagnose and stage ampullary tumours are lacking and current advice is predominantly based on guidelines for patients with pancreatic cancer. Despite many similarities with pancreatic cancer, the location and nature of ampullary tumours necessitates specific data. Current pancreatic cancer guidelines are not consistent. In the ESMO guidelines, CT is recommended in all patients, and EUS with fine-needle aspiration and biopsy in case of doubt^7^. The European Society of Gastrointestinal Endoscopy guideline^13^ only recommends endoscopic assessment with biopsy.

In daily clinical practice, the proper treatment for ampullary tumours is preferably selected based on pathological, local, and regional assessment of the tumour. CT provides information on the tumour and involvement of nearby structures, lymph node(s), and distant metastases^37^. The reported sensitivity of CT for assessing the nature of ampullary tumours in the two included studies^29,32^ is highly variable. Sample sizes were small, and different methods were used for pathology sampling and image acquisition^29^. EUS and IDUS are valuable for describing local infiltration of the tumour and provide an opportunity for biopsy. Lymph node involvement or metastases cannot be assessed. The sensitivity and specificity reported in the included studies for EUS and IDUS are higher than those for CT, and several studies^38–40^ have shown the advantage in tumour classification. Specific data on the diagnostic accuracy of MRI or nuclear imaging are scarce and should be the subject of future research. The value of periodic imaging follow-up as a diagnostic modality was not investigated in the studies included in the present review. Reliable follow-up was especially relevant for patients who underwent local resection or who were not good candidates for endoscopic or surgical resection^13^.

This review had several limitations. Most studies included were retrospective, had a moderate-to-high risk of bias and were published more than 10 years ago. Diagnostic modalities evolve rapidly and newer ones might nowadays be better at discriminating ampullary tumours. No RCTs were available on this subject. Limited data and heterogeneity precluded meta-analysis.

The limited number of included studies and their quality highlights the need for continued research on this topic. Data from registries and prospective cohorts are needed to design clinical trials to further assess the best diagnostic approach. Currently, no studies have been registered at ClinicalTrials.gov or the International Clinical Trials Registry Platform. Recently, an international registry was initiated on the treatment of ampullary tumours by the International Study Group on Ampullary Cancers^41^, which will provide more information regarding the use and accuracy of diagnostic modalities in daily clinical practice. The main challenge for future studies is to have an adequate sample size. (Inter)national collaboration should be encouraged. Histological confirmation of the diagnosis in biopsies and/or resection specimens of malignant and benign tumours is needed to assess the diagnostic accuracy of these procedures. This will allow the development of a clear algorithm, including clinical presentation, and single and combined diagnostic modalities, for choosing the best diagnostic and treatment strategy for ampullary tumours in every patient.

## Supplementary Material

znad432_Supplementary_Data

## Data Availability

The data that support the findings in this study are available from the corresponding author upon request.
