# Supplementary material for: Diagnostic accuracy of cross-sectional and endoscopic imaging in ampullary tumours: systematic review
Source: Br J Surg. 2024 Jan 10;111(1):znad432. doi: 10.1093/bjs/znad432 (PMC10782211; doi:10.1093/bjs/znad432)
Supplement: znad432_Supplementary_Data [file znad432_supplementary_data.docx]

**Diagnostic accuracy of cross-sectional and endoscopic imaging in ampullary tumours: systematic review**

Anouk J. de Wilde^1*^, Evelien J.M. de Jong^2*^, Kurinchi S. Gurusamy^3^, Mohammad Abu Hilal^4^, Marc G. Besselink^5,6^, Maxime J.L. Dewulf^1^, Sandra M.E. Geurts^2^, Ulf P. Neumann^1^, Steven W.M. Olde Damink^1^, Jan-Werner Poley^7^, Vivianne C.G. Tjan-Heijnen^2^, Judith de Vos-Geelen^2^, Georg Wiltberger^8^, Mariëlle M.E. Coolsen^1^ & Stefan A.W. Bouwense^1^

^*^Shared first authorship

**Affiliations**

^1^Department of Surgery, Maastricht University Medical Center+, Maastricht, The Netherlands

^2^Department of Internal Medicine, Division of Medical Oncology, Maastricht University Medical Center+, GROW, Maastricht University, Maastricht, The Netherlands

^3^Division of Surgery and Interventional Science, Royal Free Campus, UCL, London, United Kingdom

^4^Department of Surgery, Fondazione Poliambulanza, Brescia, Italy

^5^Amsterdam UMC, location University of Amsterdam, Department of Surgery, Amsterdam, the Netherlands

^6^Cancer Center Amsterdam, the Netherlands

^7^Department of Gastroenterology and Hepatology, Maastricht University Medical Center+, Maastricht, The Netherlands

^8^Department of General, Visceral, and Transplantation Surgery, University Hospital of RWTH Aachen, Aachen, Germany.

**Correspondence**

Anouk de Wilde, Department of Surgery, Maastricht University Medical Center+, P. Debyelaan 25, 6229 HX Maastricht, The Netherlands. E-mail: [anouk.de.wilde@mumc.nl](mailto:anouk.de.wilde@mumc.nl)

**Supplementary Materials - Index**

| **Supplementary Appendixes** |  |
| --- | --- |
| 1. Search Pubmed | *pag. 3* |
| 1. Search in CINAHL 2. Search in Cochrane 3. Search in Embase | *pag. 4  pag. 5 pag. 6* |
|  |  |
| **Supplementary Figures and Tables** |  |
| Table 2 - Quality analysis of included studies: Quality Assessment of Diagnostic Accuracy Studies-2 and Quality Assessment of Diagnostic Accuracy Studies-Comparative (patient selection (P), index test (I), reference test (R), flow and timing (FT)) | *pag. 8* |
| Table 3b - Summary of the diagnostic test accuracy of the conference abstracts | *pag. 10* |
|  |  |

**Supplementary Appendixes**

1. **Search in Pubmed**

(((((((((("Ampulla of Vater"[Mesh]) OR (Vater ampulla)) OR (Ampulla of vater)) OR (Ampullary)) OR (Hepatopancreatic ampulla)) OR (Vater* ampulla)) OR (Major duodenal papilla)) OR (Greater duodenal papilla)) AND (((((((("Neoplasms"[Mesh]) OR ("Common Bile Duct Neoplasms"[Mesh])) OR (Neoplas*)) OR (Tumor*)) OR (Tumour*)) OR (Cancer*)) OR (Malignan*)) OR (Carcinom*))) AND ((((((((((((((((((((("Magnetic Resonance Imaging"[Mesh]) OR ("Ultrasonography"[Mesh])) OR ("Endoscopy, Digestive System"[Mesh])) OR ("Tomography, X-Ray Computed"[Mesh])) OR (Transabdominal ultrasound)) OR (CT scan)) OR (CT)) OR (Computed tomography)) OR (MRI)) OR (Magnetic resonance imaging)) OR (Magnetic resonance cholangiopancreatography)) OR (MRCP)) OR (Endoscopic ultrasound)) OR (EUS)) OR (Endoscopic retrograde cholangiopancreatography)) OR (ERCP)) OR (Endoscopy)) OR (Duodenoscopy)) OR ("Radionuclide Imaging"[Mesh])) OR (pet ct)) OR (nuclear scintigraphy))) AND ((((("Biopsy"[Mesh]) OR (biopsy)) OR (Surgical biopsy)) OR (Endoscopic biopsy)) OR (Cytology))

| # | Query | Results |
| --- | --- | --- |
| S1 | (MH “Magnetic Resonance Imaging”) | 126,550 |
| S2 | (MH “Ultrasonopgrahy+”) OR (MH “Endosonography”) | 104,216 |
| S3 | (MH “Endoscopy, Digestive System+”) | 27,849 |
| S4 | (MH “Tomography, X-ray Computed+”) | 109,718 |
| S5 | (MH “Cholangiopancreatography, Endoscopic Retrograde”) | 4,176 |
| S6 | transabdominal ultrasound | 437 |
| S7 | ct scan | 46,267 |
| S8 | computed tomography | 150,525 |
| S9 | MRI | 85,895 |
| S10 | magnetic resnonace imaging | 144,829 |
| S11 | magnetic resonance cholangiopancreatography | 446 |
| S12 | MRCP | 506 |
| S13 | ERCP | 3,446 |
| S14 | endoscopic retrograde cholangiopancreatography | 4,706 |
| S15 | endoscopic ultrasound | 4,419 |
| S16 | EUS | 2,991 |
| S17 | Endoscopy | 37,570 |
| S18 | Duodenoscope | 257 |
| S19 | S1 or S2 or S3 or S4 or S5 or S6 or S7 or S8 or S9 or S10 or S11 or S12 or S13 or S14 or S15 or S16 or S17 or S18 | 414,067 |
| S20 | (MH “Biopsy+”) | 47,714 |
| S21 | Biopsy | 78,361 |
| S22 | surgical biopsy | 1,651 |
| S23 | endoscopic biopsy | 980 |
| S24 | Cytology | 6,873 |
| S25 | S20 or S21 or S22 or S23 or S24 | 83,378 |
| S26 | Ampulla of vater | 208 |
| S27 | Ampullary | 488 |
| S28 | Vater* ampulla | 209 |
| S29 | Major duodenal papilla | 44 |
| S30 | S26 or S27 or S28 or S29 | 687 |
| S31 | (MH “Duodenal Neoplasms”) | 676 |
| S32 | Neoplas* | 494,148 |
| S33 | Tumor* | 239,373 |
| S34 | Tumour* | 30,251 |
| S35 | Cancer* | 461,807 |
| S36 | Malignan* | 76,543 |
| S37 | Carcinom* | 111,884 |
| S38 | S31 or S32 or S33 or S34 or S35 or S36 or S37 | 736,452 |
| S39 | S19 AND S25 AND S30 AND S38 | 67 |

1. **Search in CINAHL**

*Limiters/Expanders: Expanders – Apply equivalent subjects; Serach modes – Boolean/Phrase*

*Last Run Via: Interfase – EBSCOhost Research Databases; Search Screen – Advanced Search; Database - CINAHL*

1. **Search in Cochrane**

| #1 | MeSH descriptor: [Ampulla of Vater] explode all trees |
| --- | --- |
| #2 | (Ampulla of vater):ti,ab,kw |
| #3 | (Ampullary):ti,ab,kw |
| #4 | (“major duodenal papilla”):ti,ab,kw |
| #5 | #1 or #2 or #3 or #4 |
| #6 | MeSH descriptor [Duodenal Neplasms] explode all trees |
| #7 | (Neoplasms):ti,ab,kw |
| #8 | (Tumor):ti,ab,kw |
| #9 | (Tumour):ti,ab,kw |
| #10 | (Cancer):ti,ab,kw |
| #11 | (Carcinoma):ti,ab,kw |
| #12 | (malignancy):ti,ab,kw |
| #13 | #6 or #7 or #8 or #9 or #10 or #11 or #12 |
| #14 | #5 and #13 |
| #15 | MeSH descriptor: [Magnetic Resonance Imgaging] explode all trees |
| #16 | MeSH descriptor: [Ultrasonography] explode all trees |
| #17 | MeSH descriptor: [Endoscopy, Digestive System] explode all trees |
| #18 | MeSH descriptor: [Tomography, X-Ray Computed] explode all trees |
| #19 | MeSH descriptor: [Cholangiopancreatography, Endoscopic Retrograde] explode all trees |
| #20 | MeSH descriptor: [Endosonography] explode all trees |
| #21 | (“transabdominal ultrasonography”):ti,ab,kw |
| #22 | (CT scan): ti,ab,kw |
| #23 | (“computed tomography scan”):ti,ab,kw |
| #24 | (“MRI scan”):ti,ab,kw |
| #25 | (“magnetic resonance imgaging”):ti,ab,kw |
| #26 | (“magnetic resonance cholangio-pancreatography”):ti,ab,kw |
| #27 | (MRCP):ti,ab,kw |
| #28 | (ERCP):ti,ab,kw |
| #29 | (“endoscopic retrograde cholangio-pancreatography”):ti,ab,kw |
| #30 | (endoscpic ultrasound):ti,ab,kw |
| #31 | (“EUS”):ti,ab,kw |
| #32 | (“endoscope”):ti,ab,kw |
| #33 | (duodenoscope):ti,ab,kw |
| #34 | #15 or #16 or #17 or #18 or #19 or #20 or #21 or #22 or #23 or #24 or #25 or #26 or #27 or #28 or #29 or #30 or #31 or #32 or #33 |
| #35 | #14 and #34 |
| #36 | MeSH descriptor: [Biopsy] explode all trees |
| #37 | (“biopsy”):ti,ab,kw |
| #38 | (surgical biopsy):ti,ab,kw |
| #39 | (endoscopic biopsy):ti,ab,kw |
| #40 | (“cytology”):ti,ab,kw |
| #41 | #36 or #37 or #38 or #39 or #40 |
| #42 | #35 and #41 |

1. **Search in Embase**

| 1. | exp Vater papilla carcinoma/ or exp ämpulla of Vater”/ or exp Vater papilla/ |
| --- | --- |
| 2. | Vater ampulla.mp. |
| 3. | Ampulla of vater.mp. |
| 4. | Ampullary.mp. |
| 5. | Hepatopancreatic ampulla.mp. |
| 6. | Vater* ampulla.mp. |
| 7. | Hepatopancreatic duct.mp. |
| 8. | 1 or 2 or 3 or 4 or 5 or 6 or 7 |
| 9. | exp neoplasm/ |
| 10. | Neoplas*.tw. |
| 11. | Tumor*.tw. |
| 12. | Tumour*.tw. |
| 13. | Cancer*.tw. |
| 14. | Malignan*.tw. |
| 15. | Carcinom*.tw. |
| 16. | 9 or 10 or 11 or 12 or 13 or 14 or 15 |
| 17. | 8 and 16 |
| 18. | exp neuclear magnetic resonance imaging/ |
| 19. | exp echography/ |
| 20. | exp biliary tract endoscopy/ or exp digestive tract endoscopy/ |
| 21. | exp x-ray computed tomography |
| 22. | exp endoscopic retrograde cholangiopancreatography/ |
| 23. | exp endoscopic ultrasonography/ |
| 24. | transabdominal ultrasound.mp. |
| 25. | CT-scan.mp. |
| 26. | CT.mp. |
| 27. | Computed tomography.mp. |
| 28. | MRI.mp. |
| 29. | Magnetic resonance imaging.mp. |
| 30. | Magnetic resonance cholangiopancreatography.mp. |
| 31. | MRCP.mp. |
| 32. | Endoscopic ultrasound.mp. |
| 33. | EUS.mp. |
| 34. | Endoscopic retrograde cholangiopancreatography.mp. |
| 35. | ERCP.mp. |
| 36. | Endoscopy.mp. |
| 37. | Duodenoscopy.mp. |
| 38. | 18 or 19 or 20 or 21 or 22 or 23 or 24 or 25 or 26 or 27 or 28 or 29 or 30 of 31 or 32 or 33 or 34 or 35 or 36 or 37 |
| 39. | 17 and 38 |
| 40. | exp endoscopic biopsy brush/ or exp endoscopic ultrasound guided fine needle biopsy/ or exp endoscopic biopsy/ or exp endoscopic biopsy needle/ or exp biopsy brush/ |
| 41. | Biop*.tw. |
| 42. | Surigcal biop*.tw. |
| 43. | Endoscopic biop*.tw. |
| 44. | Cytology.tw. |
| 45. | 40 or 41 or 42 or 43 or 44 |
| 46. | 39 and 45 |
| 47. | exp “sensitvity and specificity”/ |
| 48. | Accuracy.mp. |
| 49. | Sensitivity.mp. |
| 50. | Specificity.mp. |
| 51. | Predictiv value*.mp. |
| 52. | 47 or 48 or 49 or 50 or 51 |
| 53. | 46 and 52 |

**Supplementary Figures and Tables**

Table 1 - Quality analysis of included studies: Quality Assessment of Diagnostic Accuracy Studies-2 and Quality Assessment of Diagnostic Accuracy Studies-Comparative (patient selection (P), index test (I), reference test (R), flow and timing (FT))

| Study | Test | Risk of bias  (QUADAS-2) | | | | Applicability concerns  (QUADAS-2) | | | | | Risk of bias  (QUADAS-C) | | | |  |  |
| --- | --- | --- | --- | --- | --- | --- | --- | --- | --- | --- | --- | --- | --- | --- | --- | --- |
|  |  | **P** | **I** | **R** | **FT** |  | **P** | **I** | **R** |  | **P** | **I** | **R** | **FT** |  |  |
| Bardales et al.  1997 | Brush cytology | ✓ | ✓ | ? | ✗ |  | ✗ | ✓ | ✓ |  | NA | NA | NA | NA |  |  |
| Heinzow et al.  2011 | ETP | ✓ | ✓ | ✗ | ✗ |  | ✓ | ✓ | ✓ |  | ✓ | ? | ✗ | ✗ |  |  |
|  | IDUS | ✓ | ✓ | ✗ | ✗ |  | ✓ | ✓ | ✓ |  |  |  |  |  |  |  |
| Ito et al.  2007 | Forceps biopsy | ✓ | ✓ | ✓ | ✗ |  | ✓ | ✓ | ✓ |  | NA | NA | NA | NA |  |  |
| Manta et al. 2010 | EUS | ✗ | ✓ | ✓ | ✗ |  | ✓ | ✓ | ✓ |  | NA | NA | NA | NA |  |  |
| Menzel et al. 1999 | EUS | ✓ | ✓ | ? | ✗ |  | ✓ | ✓ | ✓ |  | ✓^*^ | ✓^*^ | ✗^*^ | ✗^*^ |  |  |
|  | IDUS | ✓ | ✓ | ? | ✗ |  | ✓ | ✓ | ✓ |  |  |  |  |  |  |  |
|  | Forceps biopsy | ✓ | ✓ | ? | ✗ |  | ✓ | ✓ | ✓ |  |  |  |  |  |  |  |
| Pongpornsup et al.  2016 | CT | ✓ | ✓ | ? | ? |  | ✓ | ✓ | ✓ |  | NA | NA | NA | NA |  |  |
| Rodríguez et al.  2002 | Biopsy during ERCP | ✗ | ✓ | ✓ | ✓ |  | ✓ | ✓ | ✓ |  | NA | NA | NA | NA |  |  |
| Sauvanet et al.  1997 | EUS | ✗ | ✓ | ✓ | ✗ |  | ✗ | ✓ | ✓ |  | ✗^*^ | ? ^*^ | ✓^*^ | ✗^*^ |  |  |
|  | SVD  Forceps biopsy | ✗  ✗ | ✓  ✓ | ✓  ✓ | ✗  ✗ |  | ✗  ✗ | ✓  ✓ | ✓  ✓ |  |  |  |  |  |  |  |
| Sperti et al. 2006 | CT  PET/CT | ✓  ✓ | ✓  ✓ | ?  ? | ✗  ✗ |  | ✓  ✓ | ✓  ✓ | ✓  ✓ |  | ✓ | ? | ✗ | ✗ |  |  |
| Wen et al. 2020 | PET/CT | ? | ✓ | ✓ | ✗ |  | ✓ | ✓ | ✓ |  | ✗ | ✓ | ✓ | ✓ |  |  |
|  | CT+MRI | ? | ✓ | ✓ | ✗ |  | ✓ | ✓ | ✓ |  |  |  |  |  |  |  |

✓ Low Risk, ✗High risk, ? Unclear risk

^*^Result for all three comparisons

Abbreviations: CT-scan = computed tomography; ERCP = endoscopic retrograde cholangiopancreatography; ETP = endoscopic transpapillary biopsy; EUS = endoscopic ultrasound; IDUS = intraductal ultrasound; MRI = magnetic resonance cholangiopancreatography; NA = not applicable; PET/CT = positron emission tomography and computed tomography; QUADAS-2 = quality assessment of diagnostic accuracy studies 2; QUADAS-C = quality assessment of diagnostic accuracy studies-comparative; SVD = side viewing duodenoscopy; US = ultrasonography.

Comment:
Risk of bias is judged as ‘low’, ‘high’, or ‘unclear’. If all domains (patient selection, index test, reference test, and flow and timing) are rated as ‘low’ then risk of bias can be judged ‘low’. If any domain is rated as ‘high’ this flags the potential for bias.

Concerns regarding applicability are rated in the similar way as the risk of bias: ‘low’, ‘high’, or ‘unclear’ but apply only to three domains (patient selection, index test, and reference test).

QUADAS-C is an extension to QUADAS-2 to assess the risk of bias in comparative diagnostic accuracy studies which compare two or more index tests in one study. Risk of bias is again judged as ‘low’, ‘high’, or ‘unclear’.

Table 2 - Summary of the diagnostic test accuracy of the conference abstracts

| Abstracts | Test | Outcomes |
| --- | --- | --- |
| Chen et al. | EUS, ERCP, CT-scan, US | EUS: Sensitivity 95%  ERCP: Sensitivity 95%  CT: Sensitivity 19%  US: Sensitivity 5%  **Overall T-staging**  EUS: Sensitivity 75%  CT: Sensitivity 5%  US: Sensitivity 0% |
| Peng et al. | EUS | Sensitivity 80%  Specificity 93% |
| Sharaiha et al. | EUS | Sensitivity 100%  Specificity 72%  **Overall T-staging**  Sensitivity 66.7%  Specificity 91.7% |

Abbreviations: CT = computed tomography; ERCP = endoscopic retrograde cholangiopancreatography; EUS = endoscopic ultrasound; US = ultrasonography.
